# Pharmacokinetic and subjective assessment of prototype JUUL2 electronic nicotine delivery system in two nicotine concentrations, JUUL system, IQOS, and combustible cigarette

**DOI:** 10.1007/s00213-022-06100-0

**Published:** 2022-02-20

**Authors:** Nicholas I. Goldenson, Erik M. Augustson, Joey Chen, Saul Shiffman

**Affiliations:** 1 Juul Labs, Inc, 1000 F Street NW, Suite 800, Washington, D.C 20004 USA; 2 Pinney Associates, Inc, Pittsburgh, PA USA

**Keywords:** Nicotine, Pharmacokinetics, Subjective effects, Electronic nicotine delivery system, Heated tobacco product, JUUL, IQOS, Cigarette

## Abstract

**Rationale:**

Electronic nicotine delivery systems and heated tobacco products are noncombustible alternatives for adult smokers. Evidence suggests sufficient nicotine delivery and satisfying effects are necessary to facilitate switching away from smoking; nicotine delivery varies across electronic nicotine delivery systems within limited nicotine concentrations.

**Objectives:**

To assess the nicotine delivery and subjective effects of prototype JUUL2 System in two nicotine concentrations, currently-marketed US JUUL System (“JUUL”), IQOS-brand heated tobacco product, and combustible cigarettes.

**Methods:**

Adult smokers (*N* = 40) completed a 5-arm cross-over product-use laboratory confinement study. Nicotine pharmacokinetics and subjective effects were assessed following use of: (1) JUUL2 prototype 18 mg/mL nicotine; (2) JUUL2 prototype 40 mg/mL; (3) JUUL 59 mg/mL; (4) IQOS 18 mg/g; and (5) usual brand combustible cigarette, each evaluated during ad libitum (10 min) and controlled (5 min, 10 standardized puffs) use.

**Results:**

Nicotine delivery was greatest for combustible cigarettes, followed by JUUL2 prototype 40 mg/mL, IQOS, JUUL2 prototype 18 mg/mL, and JUUL 59 mg/mL. Nicotine delivery from JUUL2 prototype 18 mg/mL was significantly greater than JUUL 59 mg/mL after ad libitum use. JUUL products were significantly more satisfying and effective at reducing craving than IQOS. JUUL2 prototype 40 mg/mL was significantly more aversive than other JUUL products.

**Conclusions:**

Prototype JUUL2 and JUUL 59 mg/mL products were rated higher than IQOS on subjective measures associated with switching away from smoking. The JUUL2 prototype 40 mg/mL produced aversive responses and would require modifications to be a viable product for adult smokers. Nicotine delivery and subjective responses to JUUL2 prototype 18 mg/mL suggest a product based on this prototype may facilitate increased switching among adult smokers.

**Supplementary Information:**

The online version contains supplementary material available at 10.1007/s00213-022-06100-0.

## Introduction

The harms of cigarette smoking primarily result from exposure to the toxicants and carcinogens produced by the combustion of tobacco, rather than from nicotine—the principal constituent that maintains smoking (Benowitz 2010; Gottlieb and Zeller 2017; USDHHS 2014). Noncombustible alternative nicotine-delivery products such as electronic nicotine delivery systems (ENDS) and heat-not-burn or heated tobacco products (HTP) have the potential to benefit public health by helping smokers who would not otherwise quit to switch completely away from cigarettes (Gottlieb and Zeller 2017). Although both ENDS and HTP deliver nicotine without combusting tobacco, and thus expose smokers to lower levels of harmful chemicals than cigarettes (Akiyama and Sherwood 2021), they use distinct technologies: ENDS aerosolize an e-liquid (typically a mixture of glycerol and/or propylene glycol) containing nicotine (NASEM 2018), whereas HTP typically heat tobacco leaf to a temperature below that required to combust tobacco but sufficient to release a nicotine-containing aerosol (Mallock et al. 2019).

Prior studies have compared the JUUL system ENDS (“JUUL”) and IQOS HTP, with mixed results: a recent study of smokers who had almost completely transitioned to ENDS found that the nicotine delivery of JUUL 59 mg/mL exceeded that of IQOS and that JUUL reduced craving for cigarettes more effectively than IQOS (Phillips-Waller et al. 2021). In contrast, two previous studies did not find significant differences in nicotine delivery or subjective effects between JUUL and IQOS among adult smokers who did not use ENDS (Buchhalter et al. 2020; Maloney et al. 2020). Hence, it is unclear if differences exist in the nicotine PK and subjective effects of JUUL and IQOS.

Public health authorities and regulatory agencies recognize that noncombustible nicotine-delivery products must effectively deliver nicotine and produce satisfying effects to successfully convert adult smokers (Abrams et al. 2018a, 2018b; FDA 2019). Recent data supports this concept: a randomized clinical trial that manipulated ENDS nicotine concentration found that smokers assigned to the highest nicotine concentration (36 mg/mL) experienced the greatest reduction in cigarette smoking and concomitant exposure to smoking-related toxicants, and the authors noted that ENDS must deliver sufficient nicotine to facilitate switching among smokers (Cobb et al. 2021). Consistent with this, an observational study (Goldenson et al. 2021a) found that adult smokers in the UK using JUUL with nicotine concentrations below 20 mg/mL mandated by the European Union Tobacco Products Directive (EU 2014) were less likely to switch away from smoking than US and Canadian smokers using JUUL with 59 or 35 mg/mL nicotine concentration with demonstrated higher nicotine delivery (Goldenson et al. 2021b).

ENDS are a highly heterogeneous category of products, consisting of both closed systems and open systems with a wide range of customizable options: the nicotine delivery of ENDS is influenced by a combination of factors beyond the nicotine concentration in the e-liquid, including device characteristics that affect aerosol production and user behavior (Jacobson et al. 2021). Given the importance of sufficient nicotine delivery in supporting switching away from smoking, research has explored factors other than nicotine concentration that may modulate nicotine delivery from ENDS and subjective effects.

ENDS with greater device power produce more aerosol, deliver more nicotine, and result in more rewarding subjective effects (Floyd et al. 2018; Hiler et al. 2020; Kosmider et al. 2018; Leventhal et al. 2020; Peace et al. 2018; Talih et al. 2014). A controlled laboratory study that manipulated several ENDS device and e-liquid characteristics found that ENDS that produce greater aerosol mass more effectively delivered nicotine and were rated significantly higher on measures of product liking (Ebajemito et al. 2020). Accordingly, the current study evaluated prototype JUUL2 ENDS products that aerosolize more e-liquid and concomitantly deliver greater aerosol mass and nicotine, with the aim of producing a usage experience that facilitates smokers switching away from cigarettes.

The primary aims of the current residential laboratory study were to (1) evaluate the nicotine PK and subjective effects of prototype JUUL2 ENDS products compared to JUUL, IQOS, and combustible cigarettes among adult smokers and (2) assess nicotine PK and subjective responses to prototype JUUL2 ENDS with two different nicotine concentrations (18 and 40 mg/mL) in order to inform development of ENDS products that could help smokers switch away from cigarettes.

## Methods

### Participants

Healthy, adult combustible cigarette smokers who were not intending to quit smoking were recruited in the Montreal, Canada, metropolitan area in 2021. Inclusion criteria were (1) 22–65 years of age; (2) cigarette smoking for ≥ 12 months prior to screening; (3) currently smoking an average of ≥ 10 nonmentholated cigarettes per day (verified by urine cotinine ≥ 200 ng/mL and exhaled carbon monoxide > 10 ppm). Exclusion criteria were (1) use of any prescription smoking cessation medications (e.g., varenicline, bupropion) within 30 days prior to study Day 1; (2) plan to quit smoking during the study or postpone a quit attempt in order to participate in the study; (3) medical (including positive COVID-19 test) or psychiatric condition that could interfere with conduct of study or jeopardize participant safety; (4) positive urine screen for drugs of abuse or positive alcohol breath test; and (5) pregnancy for females. There were no eligibility criteria regarding use of ENDS, HTP, other noncigarette tobacco products or nicotine replacement therapy.

All participants provided written informed consent and were compensated for their participation. The Advarra Institutional Review Board (https://www.advarra.com/review-services/institutional-review-board/) approved the study protocol and the study was conducted in accordance with the Declaration of Helsinki and the TriCouncil Policy Statement (Canada).

### Design

The study utilized an open-label, randomized, crossover within-subjects design. An approximately equal number of participants were randomly assigned to five product sequences based on a block randomization scheme generated from a Latin Square design.

### Procedure

Eligible participants were confined to a clinical residential research facility for the duration of the study, allowing for staff monitoring of compliance to protocol. Prior to the first day of product use, participants completed a product training and familiarization session for the JUUL products and IQOS in which they watched a training video and then used each of the JUUL and IQOS test products for 10 min ad libitum; use of successive products was separated by 15 min. Participants were also instructed how to perform the controlled puffing sequence (i.e., inhale for 3 s, remove the product from mouth and inhale for an additional 3 s before exhaling; repeated every 30 s for a total of 10 puffs [5 min total]) by watching a training video and then practicing the controlled puffing sequence using JUUL 59 mg/mL.

Participants who did not tolerate or were unwilling to use any of the study products during the product familiarization period (as determined by participant self-report or observations by study staff; e.g., excessive coughing) or were unable to successfully perform the controlled puffing sequence by reducing the weight of the pod by 20–60 mg (to standardize exposure) in up to three attempts were deemed ineligible. Following completion of the familiarization period, participants were allowed to smoke their UB cigarettes ad libitum for 4 h, ending at least 12 h prior to the first day of product use (Supplementary Fig. 1).

During the five product-use days, tobacco/nicotine product use was only permitted during the ad libitum and controlled product use sessions. The experimental procedures for the product use sessions were identical on each of the five product-use days. Participants first used their randomly assigned test product during a 10-min ad libitum session (preceded by ≥ 12 h of nicotine/tobacco product abstinence) and then, at least 6 h later, during a controlled use session (10 standardized puffs) that lasted 5 min. Participants were monitored and guided by study staff during controlled and ad libitum use sessions and if necessary were assisted in performing the controlled use procedure. It is important to note the difference in the duration of the ad libitum and controlled use sessions (10 vs. 5 min) when interpreting PK parameters in these two use conditions.

In both ad libitum and controlled sessions, venous blood samples were collected 5 min before and 1.5, 3, 5, 6, 7, 8, 10, 15, 30, and 60 min after the start of product use (Supplementary Table 1). Given the longer duration of product use in the ad libitum use sessions, which was expected to result in higher nicotine levels, additional blood draws were also taken at 45, 75, and 90 min, to characterize later phases of the nicotine delivery time course. PK profiles with multiple missing blood draws were excluded.

In all sessions, subjective responses to use of study products were assessed with the modified Product Evaluation Scale (mPES; Hatsukami et al. 2013) 30 min following the start of product use, after the 30-min blood collection. In all sessions that included JUUL products, pods were weighed before and after use and mass of e-liquid aerosolized was calculated; in the ad libitum use condition, the number of cigarettes and IQOS heatsticks used was recorded. All JUUL and IQOS products were used with fully charged batteries and unused pods or heat sticks for each use session.

Participants were instructed to inform the study personnel of any adverse events (AE; any untoward medical occurrence experienced during the study); consistent with practice in human trials, AEs were spontaneously reported or elicited during open-ended questioning, examination, or evaluation. AEs were classified based on intensity (severity), seriousness, and causal relation to use of study product by a medically qualified investigator at the study site who was not employed by Juul Labs.

### Study test products

Test products included (1) JUUL2 prototype 18 mg/mL nicotine in tobacco flavor; (2) JUUL2 prototype 40 mg/mL nicotine in tobacco flavor; (3) JUUL 59 mg/mL nicotine in classic tobacco flavor that was previously commercially-marketed in the US; (4) commercially available IQOS with Birch tobacco heat sticks with 18 mg/g nicotine; and (5) UB combustible cigarette. Like JUUL, the JUUL2 prototype is a closed-system ENDS that is inhalation-actuated; does not have any user-modifiable settings, controls, or buttons; and includes a temperature control system designed to maintain a consistent operating temperature independent of puff intensity to minimize production of toxicants. The JUUL2 prototype pods contained 1.2 mL of e-liquid (compared to 0.7 mL in JUULpods) consisting of nicotine (either 18 or 40 mg/mL), propylene glycol, glycerol, benzoic acid, and flavorants (the same primary ingredients as in JUULpods). The JUUL2 prototype incorporates several new technologies including a redesigned wick, larger heater zone, improved fluid delivery system, and reformulated e-liquid. Although the wattage of JUUL2 prototype is greater than JUUL, both devices operate at similar temperatures with closed-loop temperature control.

### Measures

#### Baseline characteristics

Participants reported demographic and cigarette smoking characteristics and whether they had ever and currently (in the past 90 days) used ENDS (yes/no).

### Subjective effects

The 20-item mPES, a psychometrically validated measure of subjective responses to tobacco products (Hatsukami et al. 2013) that has previously been used to evaluate ENDS including JUUL (Gades et al. 2020; Goldenson et al. 2020a, 2020b), was answered on a 7-point response scale from 1 (“not at all”) to 7 (“extremely”). The mPES included four composite subscales: “Satisfaction” (4 items), “Psychological Reward” (5 items), “Aversion” (4 items), and “Relief” (5 items).

### Data analysis

PK parameters included baseline-adjusted maximum plasma nicotine concentration (C_max-BL_) and time to reach maximum plasma nicotine concentration (T_max_); baseline-adjusted total plasma nicotine exposure was calculated using area under the curve (AUC) at 90 min in the ad libitum sessions (AUC_0-90-BL_) and 60 min (AUC_0-60-BL_) in the controlled use sessions, respectively. A derived pharmacokinetic parameter, the slope of the initial rise in plasma nicotine levels up to C_max_, was calculated as C_max-BL_ divided by T_max_ (de Wit et al. 1992).

All statistical comparisons between test products were conducted separately for the ad libitum and controlled use conditions, as the ad libitum and controlled use conditions were of different durations and thus are not comparable. Since C_max-BL_ and AUC were not normally distributed, values were log-transformed and modeled as dependent variables in linear mixed-effects models with fixed effects of test product, sequence, and period and a random participant term. Differences in C_max-BL_ and AUC were assessed using recommended methods: geometric mean ratios between study products were calculated as back-transformed (exponentiated) least-squares ratios with 2-sided 90% CIs; statistically significant differences in C_max-BL_ and AUC between test products were indicated if the 90% confidence intervals (CIs) for geometric mean ratios did not overlap with 1.00 (FDA 2001). Differences in rate of plasma nicotine rise were tested with mixed-effects models as described above. Differences in T_max_ were tested using nonparametric Wilcoxon signed rank tests. Measures of subjective effects (mPES) were analyzed on their original assessment scales using mixed-effects models.

To test if differences in PK parameters and subjective effects between test products differed by ENDS use history, separate mixed-effects models included fixed effects of ever-ENDS use (yes/no) and a test product × ENDS use interaction term.

Data were analyzed using IBM SPSS Statistics Version 28 (IBM Corp., Armonk, NY) with alpha level set to 0.05.

## Results

### Participant accrual and sample characteristics

Out of 112 individuals screened, 40 (35.7%) met all eligibility criteria, enrolled in the study, were randomized, and completed ≥ 1 product use session. The most common reasons for ineligibility were positive urine screen for drugs of abuse or alcohol (40.7%) followed by an excluding medical or psychiatric condition (27.1%). Five participants were excluded for being unable to successfully complete the product training and familiarization sessions; PK data from 10 total sessions (7 ad libitum use and 3 controlled use) were excluded due to multiple missed blood draws (Supplementary Table 2). In the ad libitum use session, 30 participants completed and provided valid PK data for all five conditions, 4 completed four conditions, 2 completed three conditions, 2 completed two conditions, and 2 completed one condition. In the controlled use session, 32 participants completed and provided valid PK data for all five conditions, 2 completed four conditions, 2 completed three conditions, 3 completed two conditions and 1 completed one condition. Six participants (15% of enrolled) withdrew from the study prior to completing all product use sessions (resulting in a total of 13 missed sessions): three subjects withdrew their consent due to personal reasons (two after using UB cigarette [one controlled and one ad libitum] and one after controlled use of IQOS), two were withdrawn due to an AE (one after controlled use of IQOS and one after controlled use of JUUL2 prototype 40 mg/mL; see the “Safety and tolerability” section), and one was withdrawn at the discretion of the investigator and sponsor after controlled use of JUUL2 prototype 18 mg/mL (Supplementary Table 3).

The sample (mean age = 43.23 years [*SD* = 13.39]) self-reported as 25.0% female, 85.0% non-Hispanic White, 10% non-Hispanic multi-racial, 2.5% non-Hispanic Asian, and 2.5% non-Hispanic Black. On average, participants reported smoking for 15.88 years (*SD* = 14.85) and currently smoking 16.78 cigarettes per day (*SD* = 4.32); 50% had ever used ENDS but only 10% were current ENDS users.

### Nicotine pharmacokinetics

**Fig. 1:**
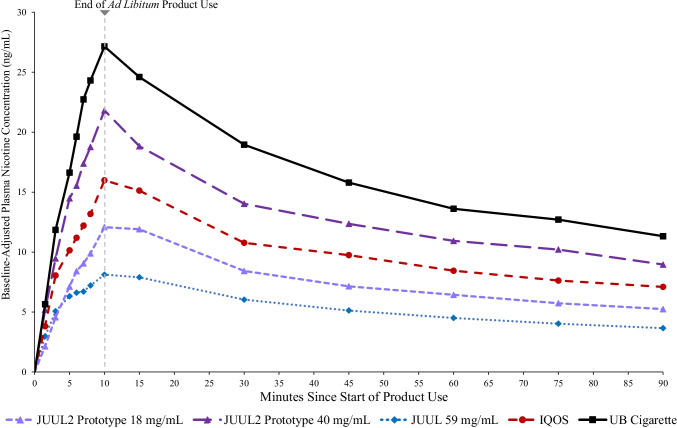
Mean baseline-adjusted plasma nicotine concentrations by nominal time in ad libitum use session. Note. JUUL2 prototype 18 mg/mL, *N* = 35; JUUL2 prototype 40 mg/mL, *N* = 36; JUUL 59 mg/mL, *N* = 36; IQOS, *N* = 38; UB cigarette, *N* = 33

**Fig. 2:**
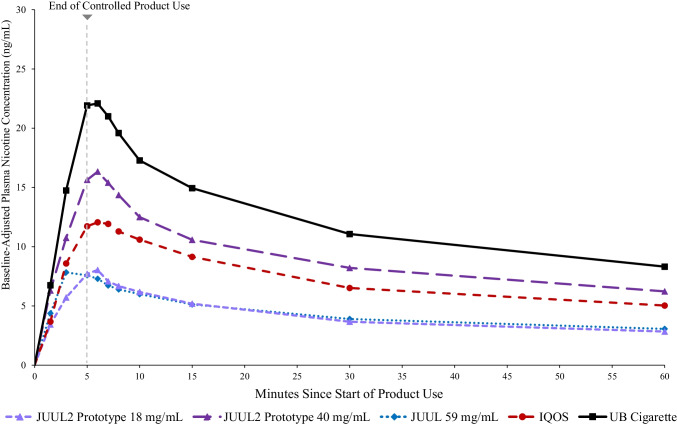
Mean baseline-adjusted plasma nicotine concentrations by nominal time in controlled use session. Note. JUUL2 prototype 18 mg/mL, *N* = 35; JUUL2 prototype 40 mg/mL, *N* = 36; JUUL 59 mg/mL, *N* = 36; IQOS, *N* = 38; UB cigarette, *N* = 33

The time courses of plasma nicotine concentrations following use of each study test product in the ad libitum and controlled use sessions over 90 and 60 min, respectively, are displayed in Figs. 1 and 2.

#### *Ad libitum* use

In the 10-min ad libitum use session, on average, participants smoked 2.2 cigarettes (*SD* = 0.49) and used 2.3 IQOS heat sticks (*SD* = 0.47); participants aerosolized 0.03 g of e-liquid when using JUUL 59 mg/mL (*SD* = 0.01), 0.14 g (*SD* = 0.06) when using JUUL2 prototype 18 mg/mL and 0.11 g (*SD* = 0.05) when using JUUL2 prototype 40 mg/mL (Supplementary Table 4). The highest mean C_max-BL_ (± *SD*) value was for UB cigarette (31.66 ± 21.70 ng/mL; Table 1), which was significantly greater than the C_max-BL_ for IQOS and all JUUL products (Table 2). C_max-BL_ for JUUL2 prototype 40 mg/mL (24.33 ± 17.97) was significantly greater than IQOS (18.22 ± 9.24 ng/mL) which was, in turn, significantly higher than JUUL2 prototype 18 mg/mL (13.98 ± 7.97 ng/mL), which was significantly higher than JUUL 59 mg/mL (9.25 ± 4.50 ng/mL; Tables 1 and 2).

**Table 1:** PK parameters of test products in controlled and ad libitum use sessions

PK parameter	JUUL 59 mg/mL	JUUL2 prototype 18 mg/mL	JUUL2 prototype 40 mg/mL	IQOS	UB cigarette
Ad Libitum Use					
C_max-BL_ (ng/mL)					
Mean (*SD*)	9.25 (4.50)^a^	13.98 (7.97)^b^	24.33 (17.97)^c^	18.22 (9.24)^d^	31.66 (21.70)^e^
Median	8.59	13.93	20.40	17.18	24.45
AUC_0-90-BL_ (ng × min/mL)					
Mean (*SD*)	478.35 (227.35)^a^	686.95 (333.93)^b^	1150.90 (632.11)^c^	892.98 (330.19)^d^	1472.84 (666.59)^e^
Median	401.57	691.44	1121.58	905.74	1370.76
Rate of Plasma Nicotine Rise (ng/mL per Minute)					
Mean (*SD*)	1.00 (0.88)^a^	1.33 (0.89)^ab^	2.59 (2.57)^c^	1.78 (1.42)^b^	3.27 (3.13)^c^
Median	0.75	1.06	1.95	1.44	2.34
T_max_ (mins)					
Mean (*SD*)	12.50 (6.06)^a^	12.15 (4.34)^a^	12.30 (7.08)^a^	15.53 (13.28)^a^	12.51 (6.79)^a^
Median	14.92	10.13	10.00	10.08	10.00
Controlled Use					
C_max-BL_ (ng/mL)					
Mean (*SD*)	9.77 (9.31)^a^	8.71 (5.08)^a^	18.42 (12.84)^b^	13.68 (5.58)^b^	24.83 (13.64)^c^
Median	7.55	7.23	14.64	12.55	22.68
AUC_0-60-BL_ (ng × min/mL)					
Mean (*SD*)	268.44 (99.18)^a^	249.96 (93.15)^a^	531.59 (260.72)^b^	433.18 (130.73)^b^	726.16 (304.70)^c^
Median	272.90	260.37	510.16	436.67	699.61
Rate of Plasma Nicotine Rise (ng/mL per Minute)					
Mean (*SD*)	2.57 (4.00)^ab^	1.48 (1.09)^a^	3.24 (3.17)^bc^	2.44 (1.66)^ab^	4.16 (3.10)^c^
Median	1.17	1.11	2.31	1.89	3.27
T_max_ (mins)					
Mean (*SD*)	6.13 (2.83)^a^	6.56 (2.04)^a^	8.02 (9.05)^a^	6.98 (2.91)^a^	9.52 (10.54)^a^
Median	6.02	6.03	6.00	6.52	6.39

*Note*. Abbreviations: *AUC*_*-BL*_ baseline-adjusted area under the curve; *C*_*max-BL*_ baseline-adjusted maximum plasma nicotine concentration; *SD* standard deviation; *T*_*max*_ time to maximum plasma nicotine concentration; *UB* usual brand

JUUL 59 mg/mL, *N* = 35; JUUL2 prototype 18 mg/mL, *N* = 37; JUUL2 prototype 40 mg/mL, *N* = 37; IQOS, *N* = 38; UB cigarette, *N* = 34

Test product means in the same row that do not share superscripts significantly differ (*p* < 0.05 or geometric mean ratio and associated 90% confidence interval does not overlap with 1.00)

**Table 2:** Geometric mean ratios of C_max-BL_ and AUC_0-90-BL_ or AUC_0-60-BL_ among test products in controlled and ad libitum use

Test product	JUUL 59 mg/mL	JUUL2 prototype 18 mg/mL	JUUL2 prototype 40 mg/mL	IQOS
	Ad libitum use—C_max-BL_ geometric mean ratio (90% *CI*)			
JUUL 59 mg/mL	—			
JUUL2 prototype 18 mg/mL	1.44 (1.22, 1.69)	—		
JUUL2 prototype 40 mg/mL	2.24 (1.91, 2.64)	1.56 (1.33, 1.84)	—	
IQOS	1.88 (1.60, 2.20)	1.31 (1.11, 1.54)	0.84 (0.71, 0.98)	—
UB cigarette	3.21 (2.72, 3.79)	2.24 (1.89, 2.65)	1.43 (1.21, 1.69)	1.71 (1.45, 2.02)
	Ad libitum use—AUC_0-90-BL_ geometric mean ratio (90% *CI*)			
JUUL 59 mg/mL	—			
JUUL2 prototype 18 mg/mL	1.36 (1.19, 1.55)	—		
JUUL2 prototype 40 mg/mL	2.18 (1.92, 2.47)	1.60 (1.41, 1.83)	—	
IQOS	1.88 (1.66, 2.13)	1.38 (1.21, 1.58)	0.86 (0.76, 0.98)	—
UB cigarette	3.11 (2.73, 3.55)	2.29 (2.01, 2.62)	1.43 (1.25, 1.63)	1.66 (1.45, 1.89)
	Controlled use—C_max-BL_ geometric mean ratio (90% *CI*)			
JUUL 59 mg/mL	—			
JUUL2 prototype 18 mg/mL	1.00 (0.82, 1.22)	—		
JUUL2 prototype 40 mg/mL	1.87 (1.54, 2.28)	1.87 (1.54, 2.27)	—	
IQOS	1.59 (1.30, 1.93)	1.58 (1.30, 1.93)	0.85 (0.70, 1.03)	—
UB cigarette	2.73 (2.23, 3.34)	2.72 (2.23, 3.33)	1.46 (1.19, 1.78)	1.72 (1.41, 2.10)
	Controlled use—AUC_0-60-BL_ geometric mean ratio (90% *CI*)			
JUUL 59 mg/mL	—			
JUUL2 prototype 18 mg/mL	0.95 (0.81, 1.12)	—		
JUUL2 prototype 40 mg/mL	1.83 (1.55, 2.15)	1.92 (1.64, 2.26)	—	
IQOS	1.64 (1.39, 1.93)	1.72 (1.46, 2.03)	0.90 (0.76, 1.05)	—
UB cigarette	2.64 (2.23, 3.12)	2.77 (2.35, 3.27)	1.44 (1.22, 1.70)	1.61 (1.37, 1.90)

*Note.* JUUL 59 mg/mL, *N* = 35; JUUL2 prototype 18 mg/mL, *N* = 37; JUUL2 prototype 40 mg/mL, *N* = 37; IQOS, *N* = 38; UB cigarette, *N* = 34

Abbreviations: *AUC*_*-BL*_ baseline-adjusted area under the curve; *CI* confidence interval, *C*_*max-BL*_ baseline-adjusted maximum plasma nicotine concentration; *UB* usual brand

Values represent geometric mean ratios (Comparator Product [Row] ÷ Test Product [Column]) and 90% CIs

Point estimates and 2-sided 90% *CI*s for the geometric mean ratios were derived from back-transformed (exponentiated) least-squares coefficients of mean product differences from mixed-effects models with fixed effects of test product, period, sequence, and participant included as a random effect

A similar pattern of results was observed for mean AUC_0-90-BL_: UB cigarette > JUUL2 prototype 40 mg/mL > IQOS > JUUL2 prototype 18 mg/mL > JUUL 59 mg/mL. Mean rate of plasma nicotine rise for UB cigarettes (3.27 ± 3.13 ng/mL per minute) did not significantly differ from JUUL2 prototype 40 mg/mL (2.59 ± 2.57 ng/mL per minute), and both were significantly greater than JUUL2 prototype 18 mg/mL (1.33 ± 0.89 ng/mL per minute), JUUL 59 mg/mL (1.00 ± 0.88 ng/mL per minute), and IQOS (1.78 ± 1.42). Rate of plasma nicotine rise of IQOS was significantly greater than JUUL 59 mg/mL but did not differ from the JUUL2 prototype 18 mg/mL. JUUL2 prototype 18 mg/mL and JUUL 59 mg/mL did not significantly differ from each other.

Mean T_max_ values for test products ranged from 12.15 to 15.53 min and did not significantly differ (*p*s > 0.37; Table 1).

#### Controlled use

In the controlled use session, on average, participants aerosolized 0.02 g of e-liquid when using JUUL 59 mg/mL (*SD* = 0.004), 0.06 g (*SD* = 0.01) when using JUUL2 prototype 18 mg/mL and 0.06 g (*SD* = 0.02) when using JUUL2 prototype 40 mg/mL (Supplementary Table 4). As in the ad libitum use session, highest mean C_max-BL_ was observed for UB cigarettes (24.83 ± 13.64 ng/mL; Table 1), which was significantly greater than all JUUL products and IQOS (Table 2). Mean C_max-BL_ for JUUL2 prototype 40 mg/mL (18.42 ± 12.84) did not significantly differ from IQOS (13.68 ± 5.58 ng/mL); both were significantly greater than JUUL2 prototype 18 mg/mL (8.71 ± 5.08 ng/mL) and JUUL 59 mg/mL (9.77 ± 9.31 ng/mL), which did not significantly differ. Similarly, mean AUC_0-60-BL_ for UB cigarettes was significantly greater than all JUUL and IQOS products. AUC_0-60-BL_ for JUUL2 prototype 40 mg/mL did not significantly differ from IQOS and both were significantly greater than JUUL 59 mg/mL and JUUL2 prototype 18 mg/mL, which did not significantly differ.

Mean rate of plasma nicotine rise for UB cigarettes (4.16 ± 3.10 ng/mL per minute) did not significantly differ from JUUL2 prototype 40 mg/mL (3.24 ± 3.17 ng/mL per minute) but was significantly greater than JUUL 59 mg/mL (2.57 ± 4.00), JUUL2 prototype 18 mg/mL (1.48 ± 1.09) and IQOS (2.44 ± 1.66). Rate of plasma nicotine rise for JUUL2 prototype 40 mg/mL was significantly greater than JUUL2 prototype 18 mg/mL, and JUUL 59 mg/mL and IQOS products did not significantly differ from each other. Mean T_max_ ranged from 6.13 to 9.52 min and did not significantly differ across products (*p*s > 0.08).

### Subjective effects

**Fig. 3:**
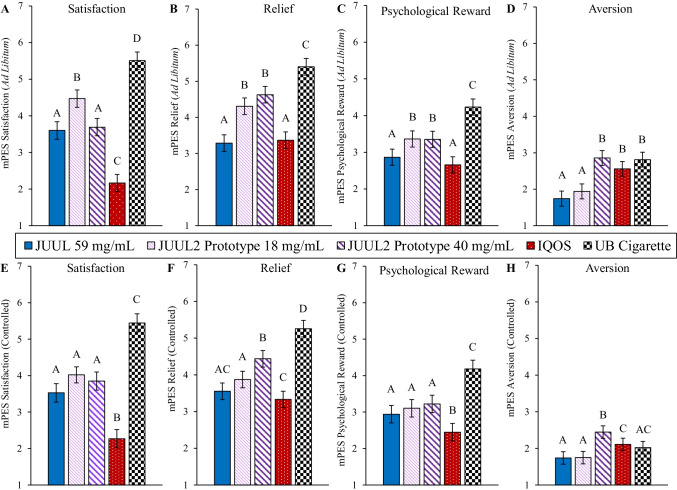
mPES composite subscale scores among test products in ad libitum and controlled use sessions (mean ± *SE*). Note. Abbreviations: mPES, modified product evaluation scale; SE, standard error. JUUL 59 mg/mL, *N* = 36; JUUL2 prototype 18 mg/mL, *N* = 37; JUUL2 prototype 40 mg/mL, *N* = 37; IQOS, *N* = 38; UB cigarette, *N* = 37. Test products that do not share the same letter significantly differ (*p* < 0.05). Values represent marginal means from mixed-effects models

Mean scores on the mPES “Satisfaction” subscale, in both ad libitum and controlled use conditions, were significantly higher for UB cigarettes than for all JUUL and IQOS products (Fig. 3A and E). In both ad libitum and controlled conditions, JUUL 59 mg/mL and both JUUL2 prototypes were rated significantly more satisfying than IQOS; in the ad libitum condition (but not controlled), the JUUL2 prototype 18 mg/mL was rated significantly more satisfying than JUUL 59 mg/mL. JUUL2 prototype 40 mg/mL and JUUL 59 mg/mL did not significantly differ in either use condition (Supplementary Table 5).

On the “Relief” subscale, mean scores for UB cigarettes was significantly higher than all other products in both ad libitum and controlled conditions (Fig. 3B and F). In the ad libitum condition, both JUUL2 prototypes were rated significantly higher than JUUL 59 mg/mL and IQOS, which did not significantly differ from each other. In the controlled use condition, both JUUL2 prototypes were rated significantly higher than IQOS, and the JUUL2 prototype 40 mg/mL was significantly greater than JUUL2 prototype 18 mg/mL and JUUL 59 mg/mL.

On the “Psychological Reward” subscale, mean scores for UB cigarettes was significantly higher than all other products in ad libitum and controlled conditions (Fig. 3C and G). In the ad libitum condition, both JUUL2 prototypes were rated significantly higher than IQOS and JUUL 59 mg/mL; IQOS and JUUL 59 mg/mL did not significantly differ. In the controlled use condition, all JUUL products were rated significantly higher than IQOS.

On the “Aversion” subscale, in the ad libitum use condition, mean scores for JUUL 59 mg/mL and JUUL2 prototype 18 mg/mL were significantly lower than JUUL2 prototype 40 mg/mL, IQOS and UB cigarette, which did not significantly differ from each other (Fig. 3 D). In the controlled use condition, JUUL2 prototype 40 mg/mL was rated significantly more aversive than all other test products; JUUL 59 mg/mL and JUUL2 prototype 18 mg/mL were rated significantly lower than IQOS (Fig. 3H).

### Effect of ENDS use history on nicotine PK and subjective effects

In the ad libitum and controlled use conditions, the test product × ENDS use interaction term was not significant for any of the PK parameters (*p*s > 0.31). Only one significant difference was observed in subjective effect outcomes between test products by ends use history: in the ad libitum use condition, the test product × ENDS use interaction term was significant for Psychological Reward (*p* = 0.01) but not any of the other subscales (*p*s > 0.12; Supplementary Table 6); in the controlled use condition, the test product × ENDS use interaction term was not significant for any of the mPES subscales (*p*s > 0.31).

### Safety and tolerability

There were no serious AEs reported in this study (Supplementary Table 7). All AEs were considered mild or moderate except for one severe AE (syncope) in the UB cigarette condition during ad libitum use (Supplementary Tables 8–9). Two participants were discontinued due to AEs: one after using JUUL2 prototype 40 mg/mL (allergic reaction; mild severity and judged possibly-related to product use; controlled use; second product used) and one after using IQOS (infected insect bites; mild severity and judged not related to product use; controlled use; first product used; Supplementary Table 3). The largest proportion of participants reported an AE after use of JUUL2 prototype 40 mg/mL (32.4%), followed by UB cigarette (29.7%), JUUL 59 mg/mL (19.4%), IQOS (15.8%) and JUUL2 prototype 18 mg/mL (13.5%). The proportion of AEs considered possibly or likely related to product use was highest for the JUUL2 prototype 40 mg/mL (32.4%) followed by UB cigarette (21.6%), IQOS (10.5%), JUUL (5.6%), and JUUL2 Prototype 18 mg/mL (5.4%).

The most commonly reported AEs were dizziness (27.5% of all participants), nausea (15.0%), procedural dizziness related to blood draw (10.0%), vomiting (7.5%), and cough (7.5%). These AEs were most reported in the JUUL2 prototype 40 mg/mL condition (12 reports of these symptoms); they were less commonly reported in other conditions (UB cigarette, 7 reports; IQOS, 7 reports; JUUL 59 mg/mL, 2 reports; JUUL2 prototype 18 mg/mL, 2 reports).

## Discussion

In this laboratory study, nicotine delivery was greatest for UB cigarettes, followed by use of JUUL2 prototype 40 mg/mL, IQOS, JUUL2 prototype 18 mg/mL, and JUUL 59 mg/mL, in that order. None of the JUUL or IQOS products delivered as much nicotine or was rated as satisfying as a combustible cigarette. However, among the JUUL and IQOS test products, subjective satisfaction was not always directly related to nicotine delivery: JUUL2 prototype 18 mg/mL delivered less nicotine than JUUL2 prototype 40 mg/mL and IQOS but was rated as significantly more satisfying and less aversive.

The ability of noncombustible alternative nicotine-delivery products to provide satisfying effects to adult smokers is central to facilitating switching: subjective satisfaction from ENDS use is associated with continued ENDS use and switching away from smoking (Evans et al. 2020; Gades et al. 2020; Goldenson et al. 2021c; Pearson et al. 2020). In a 1-year longitudinal study of US adult smokers who purchased JUUL, a 1-point increase in subjective ratings of satisfaction at baseline was prospectively associated with 27% greater odds of switching across the follow-up period (Goldenson et al. 2021c). Evidence from controlled laboratory studies suggests a relationship between nicotine dose and reinforcing effects (Kalman 2002; Kalman and Smith 2005), but increased nicotine is also sometimes associated with orosensory harshness and irritancy (Caldwell et al. 2012; Carstens and Carstens 2021; Hummel et al. 1992), and data on the effect of nicotine delivery from ENDS on subjective satisfaction is mixed (Dawkins et al. 2018; Leventhal et al. 2019; Maloney et al. 2019). Although JUUL 59 mg/mL delivered significantly less nicotine than IQOS, it was rated significantly higher on mPES “Satisfaction” subscale and lower on “Aversion” subscale; similarly, the JUUL2 prototype 18 mg/mL was rated as more satisfying and less aversive than JUUL2 prototype 40 mg/mL, despite delivering less nicotine.

Regulatory initiatives that limit the maximum nicotine concentration in ENDS to 20 mg/mL, such as the European Union Tobacco Products Directive, state that this concentration allows for nicotine delivery that is comparable to the amount of nicotine derived from smoking a combustible cigarette (EU 2014). PK data demonstrates that the original JUUL product with 18 mg/mL delivers approximately one-fifth of the nicotine delivered by a cigarette and that JUUL 59 mg/mL, compared to 18 mg/mL, more effectively reduces withdrawal symptoms and craving for cigarettes (Goldenson et al. 2021b; Phillips-Waller et al. 2021). Accordingly, it was concluded that the 18 (vs. 59) mg/mL JUUL product may have more limited potential in helping heavier and dependent adult smokers switch away from smoking (Goldenson et al. 2021b; Phillips-Waller et al. 2021). Consistent with this, an observational comparative study of JUUL users in the UK (who predominantly used 18 mg/mL) and users in the US and Canada (predominantly 59 mg/mL users), matched on demographics and smoking profile, showed that the odds of switching were approximately 80% higher among adult smokers using the higher-nicotine-concentration JUUL product (Goldenson et al. 2021a). Thus, prior research suggests that higher nicotine delivery and greater subjective satisfaction have a material bearing on ENDS products’ ability to promote switching among adult smokers.

The association of ENDS nicotine concentration and nicotine delivery is not monotonic, as other parameters such as aerosol volume moderate the relation (Benowitz et al. 2021; Jacobson et al. 2021). Consistent with their design, the JUUL2 prototypes evaluated in this study produced significantly greater aerosol mass than JUUL 59 mg/mL. In the ad libitum use condition, the JUUL2 prototype 18 mg/mL delivered significantly more nicotine than the lower-aerosol JUUL 59 mg/mL but less nicotine than a cigarette. JUUL2 prototype 18 mg/mL was also rated as more satisfying and more effective at reducing cigarette craving and withdrawal symptoms than JUUL 59 mg/mL and IQOS, but lower than a cigarette. Hence, an ENDS product based on JUUL2 prototype 18 mg/mL may facilitate increased switching among adult smokers.

PK and subjective data from laboratory studies indicates that the abuse liability of JUUL 59 mg/mL is lower than combustible cigarettes (Goldenson et al. 2020a, 2020b; Maloney et al. 2020). Additionally, real-world longitudinal evidence demonstrates that among smokers who switch to JUUL, levels of JUUL dependence are significantly lower than smokers’ prior cigarette dependence (Leavens et al. 2021; Shiffman et al. 2021). The PK and subjective effect profiles observed herein indicate that the pharmacological abuse liability of all of the JUUL and IQOS products evaluated is lower than that of cigarettes. Given that indices of product appeal commonly used to characterize abuse liability, such as subjective satisfaction, are also important for facilitating switching away from smoking (Evans et al. 2020; Goldenson et al. 2021c), some degree of abuse liability is deemed necessary for noncombustible products to successfully compete with cigarettes (Abrams et al. 2018a, 2018b; FDA 2019).

The JUUL2 prototype 40 mg/mL was rated significantly more aversive than both JUUL 59 mg/mL and JUUL2 prototype 18 mg/mL. Further, the adverse event data indicate a trend towards more dizziness and nausea for the JUUL2 prototype 40 mg/mL. These findings suggest that, as currently designed and formulated, JUUL2 prototype 40 mg/mL is not an optimal product for adult smokers. Further evolution and modification of this prototype will be needed to achieve a favorable product profile to support switching. In contrast, the JUUL2 18 mg/mL prototype produced notably greater satisfaction and less aversion, suggesting the potential to be refined into a product with limited nicotine concentration that could be useful for helping smokers switch away from cigarettes.

Strengths of the study include the evaluation of commonly used HTP and ENDS products, randomized within-subjects design, inclusion of both ad libitum and controlled use procedures and confinement of participants to a clinical laboratory setting to monitor and control nicotine/tobacco product use. The controlled use procedure facilitated standardized comparisons among the study test products by controlling puff frequency and duration, whereas the ad libitum procedure allowed participants to control their use topography and thus is likely more ecologically valid, but is not necessarily indicative of longer-term real-world use.

As is standard in PK research, this experimental study was conducted in a tightly controlled setting, and future research is needed to evaluate subjective responses to use of JUUL2 in real-world settings. However, the acute nicotine delivery and subjective effect parameters assessed in this study have been shown to be associated with real-world product use and switching behavior. Additional limitations include the open-label design which may have allowed pre-existing expectations to affect subjective responses; as in other clinical studies the exclusion criteria (e.g., drugs of abuse, medical/psychiatric conditions) may limit the generalizability of the findings. Experience with HTP use was not assessed, and ENDS use history was characterized in a limited manner, but analyses did not suggest that history of prior ENDS use moderated either PK parameters or subjective responses. Additionally, the study only assessed JUUL2 prototypes with tobacco flavors, and future research is needed to assess the PK and subjective effects of non-tobacco flavors when used in similar products. Furthermore, the JUUL2 products evaluated were developmental prototypes, and may differ from ENDS products marketed to smokers.

## Conclusions

In this sample of adult smokers, all evaluated JUUL and IQOS products delivered less nicotine than UB cigarettes. IQOS delivered more nicotine than JUUL2 prototype 18 mg/mL and JUUL 59 mg/mL, but JUUL products were generally rated as more satisfying and more effective at reducing craving than IQOS—the JUUL2 prototype 18 mg/mL and JUUL 59 mg/mL were also less aversive than IQOS. Use of JUUL 59 mg/mL and JUUL2 prototype 18 mg/mL was well tolerated under both use conditions, whereas the JUUL2 prototype 40 mg/mL generated some aversive responses. JUUL2 prototype 18 mg/mL may provide a basis for future ENDS products that can facilitate increased switching among adult smokers.

## Supplementary Information

Below is the link to the electronic supplementary material.

Supplementary file1 (PDF 195 KB)
