# Supplementary material for: Pharmacokinetic and subjective assessment of prototype JUUL2 electronic nicotine delivery system in two nicotine concentrations, JUUL system, IQOS, and combustible cigarette
Source: Psychopharmacology (Berl). 2022 Feb 20;239(3):977–88. doi: 10.1007/s00213-022-06100-0 (PMC8858085; doi:10.1007/s00213-022-06100-0)
Supplement: Supplementary file 1 — Supplementary file1 (PDF 195 KB) [file 213_2022_6100_MOESM1_ESM.pdf]

Supplementary Material File

Article Title: Pharmacokinetic and Subjective Assessment of Prototype JUUL2 Electronic Nicotine Delivery System in Two Nicotine Concentrations, JUUL System, IQOS and Combustible Cigarette

Journal Name: *Psychopharmacology*

Authors and Affiliations: Nicholas I. Goldenson, PhD,[a] Erik M. Augustson, PhD, MPH,[a] Joey Chen, BS,[a] Saul Shiffman, PhD[b]

[a]Juul Labs, Inc.
[b]PinneyAssociates, Inc.

Corresponding Author: Nicholas I. Goldenson, Juul Labs, Inc., 1000 F Street NW, Washington, D.C., 20004, e-mail: nicholas.goldenson@juul.com

Supplementary Figure 1. Schematic of Experimental Design

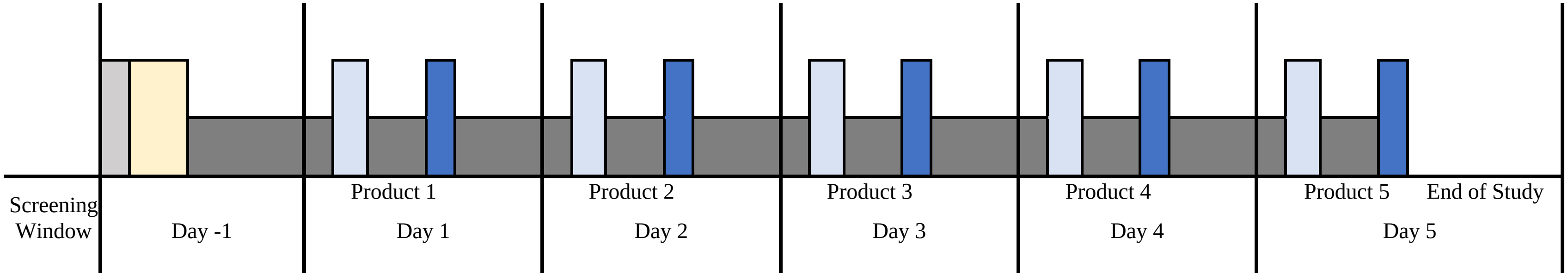

= Product training and familiarization

= 4-hour *ad libitum* cigarette smoking

= Washout Period (Nicotine/tobacco product abstinence: 12 hours before start of next day's product use or 6 hours between *ad libitum* and controlled use)

= *Ad libitum* test product use (10 minutes)

= Controlled test product use (10 puffs; 5 minutes)

Supplementary Table 1. Timeline of Blood Sampling Relative to Test Product Use

Product Use	Time of Blood Sampling in Relation to Start of Test Product Use (Minutes)														
	-5	0	1.5	3	5	6	7	8	10	15	30	45	60	75	90
Controlled	✓		✓	✓	✓	✓	✓	✓	✓	✓	✓		✓		
Ad Libitum	✓		✓	✓	✓	✓	✓	✓	✓	✓	✓	✓	✓	✓	✓

Note. Abbreviations: ENDS, electronic nicotine delivery system.

= Test Product Use

Supplementary Table 2. PK Session Data in *Ad Libitum* and Controlled Use Conditions

Use Condition	PK Sessions	JUUL 59 mg/mL N (%)	JUUL2 Prototype 18 mg/mL N (%)	JUUL2 Prototype 40 mg/mL N (%)	IQOS N (%)	UB Cigarette N (%)
Ad Libitum	No. of PK sessions completed	36 (90.0)	35 (87.5)	36 (90.0)	38 (95.0)	33 (82.5)
	No. of PK sessions excluded for missed blood draws	0 (0.0)	2 (2.5)	1 (2.5)	0 (0.0)	4 (10.0)
	No. of missing sessions due to withdrawal	4 (10.0)	3 (10.0)	3 (7.5)	2 (2.5)	3 (7.5)
Controlled	No. of PK sessions completed	35 (87.5)	37 (92.5)	37 (92.5)	38 (95.0)	34 (85.0)
	No. of PK sessions excluded for missed blood draws	1 (2.5)	0 (0.0)	0 (0.0)	0 (0.0)	2 (5.0)
	No. of missing sessions due to withdrawal	4 (10.0)	3 (7.5)	3 (7.5)	2 (2.5)	4 (10.0)

Note. Abbreviations: UB, usual brand.
N=40.

Supplementary Table 3. Reasons for Study Discontinuation in Reference to Product Use

Reason for Study Discontinuation	Last Product Used	Study Day
Voluntary withdrawal due to personal reasons	IQOS (Controlled)	4
Voluntary withdrawal due to personal reasons	UB Cigarette (*Ad Libitum*)	4
Voluntary withdrawal due to personal reasons	UB Cigarette (Controlled)	2
Adverse event (allergic reaction)	JUUL2 Prototype 40 mg/mL (Controlled)	2
Adverse event (infected insect bites)	IQOS (Controlled)	1
Withdrawn at investigator's and sponsor's discretion	JUUL2 Prototype 18 mg/mL (Controlled)	2

Note. N=6.
There were total five study days.

Supplementary Table 4. Test Product Consumption in *Ad Libitum* and Controlled Use Sessions

Use Condition	Product Consumption	JUUL 59 mg/mL	JUUL2 Prototype 18 mg/mL	JUUL2 Prototype 40 mg/mL	IQOS	UB Cigarette
Ad Libitum	No. of Cigarettes or HeatSticks, Mean (*SD*)	—	—	—	2.3 (0.47)[a]	2.2 (0.49)[a]
	Net Weight Aerosolized (g), Mean (*SD*)	0.03 (0.01)[a]	0.14 (0.06)[b]	0.11 (0.05)[c]	—	—
Controlled	No. of Cigarettes or HeatSticks, Mean (*SD*)	—	—	—	1.0 (0.0)	1.0 (0.0)
	Net Weight Aerosolized (g), Mean (*SD*)	0.02 (0.004)[a]	0.06 (0.01)[b]	0.06 (0.02)[b]	—	—

Note. JUUL, N=36; JUUL2 Prototype 18 mg/mL, N=37; JUUL2 Prototype 40 mg/mL, N=37; IQOS, N=38; UB Cigarette, N=36-37.
Net Weight Aerosolized (g) = [Pre-Weight (g) - Post-Weight (g)].
Test product means in the same row that do not share superscripts significantly differ ($p<0.05$).

Supplementary Table 5. mPES Subscale Scores of Test Products in *Ad Libitum* and Controlled Use Conditions

Use Condition	mPES Subscale	JUUL 59 mg/mL Mean (*SD*)	JUUL2 Prototype 18 mg/mL Mean (*SD*)	JUUL2 Prototype 40 mg/mL Mean (*SD*)	IQOS Mean (*SD*)	UB Cigarette Mean (*SD*)
Ad Libitum	Satisfaction	3.63 (1.49)[a]	4.49 (1.43)[b]	3.72 (1.67)[a]	2.18 (1.44)[c]	5.52 (0.96)[d]
	Relief	3.29 (1.36)[a]	4.31 (1.32)[b]	4.66 (1.54)[b]	3.37 (1.51)[a]	5.39 (1.30)[c]
	Psychological	2.87 (1.36)[a]	3.37 (1.49)[b]	3.38 (1.51)[b]	2.65 (1.23)[a]	4.22 (1.46)[c]
	Aversion	1.65 (0.85)[a]	1.92 (0.85)[a]	2.81 (1.42)[b]	2.51 (1.52)[b]	2.76 (1.36)[b]
Controlled	Satisfaction	3.53 (1.49)[a]	4.06 (1.76)[a]	3.86 (1.69)[a]	2.26 (1.36)[b]	5.49 (1.18)[c]
	Relief	3.51 (1.52)[ac]	3.86 (1.42)[a]	4.43 (1.35)[b]	3.31 (1.41)[c]	5.24 (1.07)[d]
	Psychological	2.88 (1.56)[a]	3.08 (1.56)[a]	3.19 (1.71)[a]	2.42 (1.18)[b]	4.19 (1.61)[c]
	Aversion	1.69 (0.96)[a]	1.75 (1.01)[a]	2.45 (1.17)[b]	2.07 (0.93)[c]	1.97 (1.00)[ac]

Note. Abbreviations: mPES, modified Product Evaluation Scale.
JUUL, N=36; JUUL2 Prototype 18 mg/mL, N=37; JUUL2 Prototype 40 mg/mL, N=37; IQOS, N=38; UB Cigarette, N=37.
Test product means in the same row that do not share superscripts significantly differ (p<0.05).
mPES items were answered on seven-point response scales from 1 (“Not at all”) to 7 (“Extremely”).

Supplementary Table 6. Differences in mPES Subscales between Test Products by ENDS Use History

Use Condition	Outcome	Test Product	ENDS Never-Users (N=20) Mean (*SE*)	ENDS Ever-Users (N=20) Mean (*SE*)
Ad Libitum	mPES "Psychological Reward" Subscale	JUUL 59 mg/mL	2.92 (0.31)	2.81 (0.32)
		JUUL2 Prototype 18 mg/mL	2.81 (0.31)	3.92 (0.32)*
		JUUL2 Prototype 40 mg/mL	2.89 (0.31)	3.89 (0.32)*
		IQOS	2.58 (0.31)	2.73 (0.31)
		UB Cigarette	3.79 (0.31)	4.67 (0.32)*

Note. Abbreviations: mPES, modified Product Evaluation Scale.
Values represent marginal means from mixed effects models with fixed effects of product, sequence, order ever-ENDS use and a test product × ENDS use interaction term.
*Significantly greater than ENDS never-users.
mPES was answered on seven-point response scales from 1 ("Not at all") to 7 ("Extremely").
In the controlled use condition the test product × ENDS use interaction term was not significant for any of the mPES subscales (*p*s>0.31).
in the *ad libitum* use condition the test product × ENDS use interaction term was not significant for the "Satisfaction", "Aversion" or "Relief" subscales (*p*s>0.12).

Supplementary Table 7. Study Emergent Adverse Events in *Ad Libitum* and Controlled Use Conditions

	JUUL 59 mg/mL (N=36)	JUUL2 Prototype 18 mg/mL (N=37)	JUUL2 Prototype 40 mg/mL (N=37)	IQOS (N=38)	UB Cigarette (N=37)
Any SEAE	7 (19.4)	5 (13.5)	12 (32.4)	6 (15.8)	11 (29.7)
Discontinued due to SEAE	0	0	1 (2.7)	1 (2.6)	0
Serious SEAE	0	0	0	0	0
Maximum Severity[a]					
Mild	6 (16.7)	4 (10.8)	9 (24.3)	5 (13.2)	8 (21.6)
Moderate	1 (2.8)	1 (2.7)	3 (8.1)	1 (2.6)	2 (5.4)
Severe	0	0	0	0	1 (2.7)
Relation to Study Product Use[b]					
Not Related	4 (11.1)	3 (8.1)	0	2 (5.3)	3 (8.1)
Unlikely Related	1 (2.8)	0	0	0	0
Possibly Related	2 (5.6)	2 (5.4)	11 (29.7)	4 (10.5)	8 (21.6)
Likely Related	0	0	1 (2.7)	0	0
Related	0	0	0	0	0
Common Adverse Events					
Dizziness	0	0	4 (10.8)	3 (7.9)	4 (10.8)
Nausea	1 (2.8)	0	1 (2.7)	2 (5.3)	2 (5.4)
Vomiting	1 (2.8)	0	2 (5.4)	0	0
Procedural dizziness	0	1 (2.7)	2 (5.4)	1 (2.6)	1 (2.7)
Cough	0	1 (2.7)	3 (8.1)	1 (2.6)	0

Note. Abbreviations: SEAE, Study emergent adverse events.
All values represent N (%).
[a]Subcategories represent the maximum severity experienced by individual participants.
[b]Subcategories represent the strongest relation to product use experienced by individual participants.

Supplementary Table 8. Study Emergent Adverse Events in *Ad Libitum* Use Condition

	JUUL 59 mg/mL (N=36)	JUUL2 Prototype 18 mg/mL (N=37)	JUUL2 Prototype 40 mg/mL (N=37)	IQOS (N=38)	UB Cigarette (N=37)
Any SEAE	1 (2.8)	2 (5.4)	11 (29.7)	4 (10.5)	10 (27.0)
Discontinued due to SEAE	0	0	0	0	0
Serious SEAE	0	0	0	0	0
Maximum Severity[a]					
Mild	0	1 (2.7)	8 (21.6)	3 (7.9)	7 (18.9)
Moderate	1 (2.8)	1 (2.7)	3 (8.1)	1 (2.6)	0
Severe	0	0	0	0	1 (2.7)
Relation to Study Product Use[b]					
Not Related	0	1 (2.7)	0	1 (2.6)	3 (8.1)
Unlikely Related	0	0	1 (2.7)	0	0
Possibly Related	1 (2.8)	1 (2.7)	9 (24.3)	3 (7.9)	7 (18.9)
Likely Related	0	0	1 (2.7)	0	0
Related	0	0	0	0	0
Common Adverse Events					
Dizziness	0	0	4 (10.8)	3 (7.9)	4 (10.8)
Nausea	1 (2.8)	0	1 (2.7)	2 (5.3)	2 (5.4)
Vomiting	1 (2.8)	0	2 (5.4)	0	0
Procedural dizziness	0	1 (2.7)	2 (5.4)	1 (2.6)	1 (2.7)
Cough	0	0	2 (5.4)	0	0

Note. Abbreviations: SEAE, Study emergent adverse events.
All values represent N (%).
[a]Subcategories represent the maximum severity experienced by individual participants.
[b]Subcategories represent the strongest relation to product use experienced by individual participants.

Supplementary Table 9. Study Emergent Adverse Events in Controlled Use Condition

	JUUL 59 mg/mL (N=36)	JUUL2 Prototype 18 mg/mL (N=37)	JUUL2 Prototype 40 mg/mL (N=37)	IQOS (N=38)	UB Cigarette (N=37)
Any SEAE	6 (16.7)	3 (8.1)	5 (13.5)	2 (5.3)	1 (2.7)
Discontinued due to SEAE	0	0	1 (2.7)	1 (2.6)	0
Serious SEAE	0	0	0	0	0
Maximum Severity[a]					
Mild	6 (16.7)	3 (8.1)	5 (13.5)	2 (5.3)	1 (2.7)
Moderate	0	0	0	0	0
Severe	0	0	0	0	0
Relation to Study Product Use[b]					
Not Related	4 (11.1)	2 (5.4)	0	1 (2.6)	0
Unlikely Related	1 (2.8)	0	0	0	0
Possibly Related	1 (2.8)	1 (2.7)	5 (13.5)	1 (2.6)	1 (2.7)
Likely Related	0	0	0	0	0
Related	0	0	0	0	0
Common Adverse Events					
Dizziness	0	0	1 (2.7)	0	0
Nausea	0	0	0	0	0
Vomiting	0	0	0	0	0
Procedural dizziness	0	0	0	0	0
Cough	0	1 (2.7)	2 (5.4)	1 (2.6)	0

Note. Abbreviations: SEAE, Study emergent adverse events.
All values represent N (%).
[a]Subcategories represent the maximum severity experienced by individual participants.
[b]Subcategories represent the strongest relation to product use experienced by individual participants.
